# Strategies to Improve Inadequate Bowel Preparation for Colonoscopy

**DOI:** 10.3389/fmed.2019.00245

**Published:** 2019-11-08

**Authors:** Goretti Hernández, Antonio Z. Gimeno-García, Enrique Quintero

**Affiliations:** ^1^Servicio de Gastroenterología, Hospital Universitario de Canarias, Instituto Universitario de Tecnologías Biomédicas (ITB) & Centro de Investigación Biomédica de Canarias (CIBICAN), Santa Cruz de Tenerife, Spain; ^2^Departamento de Medicina Interna, Universidad de La Laguna, Santa Cruz de Tenerife, Spain

**Keywords:** inadequate bowel preparation, risk factors of bowel cleansing, quality of colonoscopy, improve bowel cleansing, bowel cleansing

## Abstract

Bowel cleansing is one of the most important parameters included in the evaluation of colonoscopy quality. The available evidence suggests that inadequate bowel preparation reduces the diagnostic yield of colorectal neoplasia and increases post-colonoscopy colorectal cancer risk. Nowadays, up to 30% of patients referred for colonoscopy have a poor bowel cleansing. Recently, several studies have analyzed the risk factors for inadequate bowel cleansing as well as the strategies to optimize bowel preparation. In this review, we have focused on summarizing the available evidence in this field.

## Introduction

Colonoscopy is the gold-standard procedure for detecting colorectal neoplastic lesions and the removal of polyps. Indeed, polypectomy has proven to decrease colorectal cancer (CRC) incidence and mortality (1). Its efficiency depends on quality indicators as the cecal intubation rate and the adenoma detection rate (ADR) which are both directly linked with the quality of bowel cleansing. Thus, inadequate bowel preparation leads to suboptimal colonoscopy effectiveness, increasing the need to repeat colonoscopies with the subsequent consumption of resources and, more importantly, increases the risk for post-colonoscopy CRC (2, 3). However, up to 30% of diagnostic colonoscopies are reported to have an inadequate bowel cleasing (4). Recently, several studies have assessed risk factors associated with an inadequate bowel preparation, suggesting that more research is warranted on new strategies to improve bowel cleansing (5). This review aimed to analyze these recommendations, placing special attention on patients that have an increased risk of inadequate bowel preparation.

## Assessment of Bowel Cleansing Quality

Bowel cleansing should be evaluated after washing and suctioning the bowel content. Thus, an adequate bowel cleansing should allow for the detection of significant colorectal lesions higher than 5 mm (2). Several bowel-preparation scales have been developed, however the Boston Bowel Preparation Scale (BBPS) has shown to be the most validated scale and has demonstrated an excellent correlation with the ADR; it has been recommended as the scale of choice in clinical practice (6). In addition, training on this scale is available online (www.cori.org/bbps). According to the BBPS, an adequate bowel cleansing is achieved when the global score is ≥ 6 points along with a score ≥ 2 in each segment of the colon. Therefore, if a colonoscopy has an inadequate bowel preparation, it should be repeated within 1 year (7, 8). The endoscopic societies have postulated that the rate of inadequate bowel preparation in an endoscopic unit should not exceed 10–15% (2, 3).

## What is the Best Colonic Cleansing Regimen?

Studies that compared a low-residue diet (LRD) with a clear liquid diet (CLD) showed a better tolerance for the former as it preserved optimal cleansing quality (9, 10). In fact, an LRD is usually recommended over a CLD [(11, 12); Table 1].

**Table 1:** Summary of meta-analysis regarding bowel preparation.

	**Reference**	**No. studies**	**Study type**	**Aim**	**Comparison group**	**Results**
Diet	Nguyen et al. (9)	9	RCT	Outcomes on patients undergoing colonoscopy	LRD vs. CLD	Adequate bowel preparations: OR 1.21; 95% CI, 0.64–2.28
						Willingness to repeat preparation: OR 1.86; 95% CI, 1.34–2.59
						Tolerability: OR 1,92; 95% CI, 1.36–2.7
						AEs: OR 0.88; 95% CI, 0.58–1.35
	Song et al. (10)	7	RCT	Efficacy in bowel preparation	LRD vs. CLD	Excellent or good bowel preparation: RR, 1.01; 95% CI, 0.91–1.13
						Tolerance RR, 1.06; 95% CI, 1.02–1.11
						Tended to repeat the same preparation RR, 1.17; 95% CI, 1.09–1.26
						Compliance: RR 0.97; 95% CI, 0.87–1.08
						AEs: RR 0.99; 95% CI, 0.88–1.12; *P* = 0.92
Split dose regimen	Bucci et al. (13)	29	RCT	1°: efficacy of colon cleansing, 2°: to assess the runaway time, rate of compliance	Split dose vs. non-split dose regimen	Adequate preparation raw rate difference: RR 0,22; 95% CI, 0.16-0.27
						The heterogeneity was caused by: the runway time (the longer, the worse the cleansing), type of diet, male sex, use of polyethylene glycol 4 L, and the Jadad score
	Martel et al. (14)	47	RCT	1°: bowel cleanliness; 2°: willingness-to-repeat, ADR or PDR, AEs, complications	Split dose vs. non-split dose regimen	Better colon cleansing: OR, 2.51; 95% CI, 1.86–3.39
						Willing to repeat: OR, 1.90; 95% CI, 1.05–3.46
						PDR: OR 0.93; 95% CI, 0.41–2.13
						ADR: OR 1.52, 95% CI, 0.69–3.32
	Avalos et al. (15)	15	RCT	1°: bowel preparation quality; 2°: willingness to repeat, tolerability/compliance, ADR, AEs	Same day vs. split dose	High quality bowel preparation: RR 0.95; 95% CI, 0.90–1.00
						ADR: RR 0.97; 95% CI, 0.79–1.20
						Willingness to repeat: RR 1.14, 95% CI, 0.96–1.36
						Tolerability: RR 1.00; 95% CI, 0.96–1.04
						Bloating: RR 0.68; 95% CI, 0.40–0.94
	Cheng et al. (16)	14	RCT	1°: bowel cleanliness (adequate or satisfactory), 2°: cecal intubation rate, ADR, willingness to repeat, AEs	Same day vs. Split-dose regimen	Bowel cleanliness: OR 0.92; 95% CI, 0.62–1,36
						Cecal intubation rate: OR 0.87, 95% CI, 0.49–1.54
						ADR: OR 0.87; 95% CI, 0.67–1.13
						Willingness to repeat: OR 1.08; 95% CI, 0.45–2.61
						AEs: OR 0.86; 95% CI 0.53–1.39
Adjuvants	Restellini et al. (17)	77	RCT	1°: bowel cleanliness (adequate), 2° : willingness to repeat, ADR	All preparations + adjuvants vs. all preparations without adjuvants	Adequate bowel preparation: OR 1.35; 95% CI, 1.02–1.78
						Willingness to repeat proportion OR 1.40; 95%; CI, 0.91–2.15
						ADR: OR 1.03; 95%; CI, 0.86–1.23
Education tools	Guo et al. (18)	8	RCT	1°: rate of adequate bowel preparation, cecal intubation rate, PDR, AEs, willingness to repeat	Enhanced instructions vs. regular instructions	Adequate bowel preparation: OR 2.35; 95% CI, 1.65–3.35
						Cecal intubation rate: OR 2.77; 95% CI, 1.73–4.42
						PDR: OR 1.25;95% CI, 0.93–1.68
						Willing to repeat: OR 1.91; 95% CI, 1.20–3.04
						AEs: OR 0.76; 95% CI, 0.54–1.07
	Chang et al. (19)	9	RCT	1°: quality of bowel preparation, 2°: PDR and need for repeat colonoscopy	Educational intervention vs. control group	Adequate bowel preparation: RR 1.22; 95 % CI 1.10–1.36
						PDR: RR1.14; 95 % CI 0.87–1.51
						Need for repeat colonoscopy: RR 0.52; 95 %CI 0.25–1.04

*RCT, randomized clinical trial; LRD, low-residue diet; CLD, clear liquid diet; AEs, adverse events, PDR, polyp detection rate*.

Several meta-analyses have demonstrated a better efficacy in the quality of bowel preparation when a split dose regimen is used [(13, 14); Table 1]. Thus, for morning-shift colonoscopies, one part of the preparation is administered the previous day and the remaining the same day of the procedure (11, 12). Indeed, the best quality of bowel cleansing is achieved when the colonoscopy is performed within 3–5 h after bowel preparation completion (20). This regimen improves adherence, willingness to repeat the preparation, and ADR with less adverse events rates (21, 22). In addition, it allows patients to keep the fasting hours required for patient's sedation according to the American Society of Anesthesia (ASA). Indeed, a prospective observational study demonstrated no significant differences in the volume of gastric residue between patients who received the bowel preparation the day before or in a split-dose regimen (23).

Two meta-analysis have also shown no significant differences in the quality of bowel preparation, tolerance and willingness to repeat the preparation between patients who received the bowel preparation in the same morning of the procedure, and those in a split-dose regimen in both morning and afternoon-shift colonoscopies [(15, 16); Table 1].

Patients who are unable to swallow could receive the bowel preparation through a nasogastric tube. Prokinetic or antiemetic agents can also be added (11).

## How Can We Improve Bowel Preparation Quality?

### Use of Adjuvants

Adjuvants such as stimulants, prokinetics, and antifoaming have been evaluated to improve the quality and adherence to bowel preparation [(17); Table 1]. However, there are discrepancies over their use between American and European Societies of Gastrointestinal Endoscopy. The ASGE does not recommend the use of adjuvants whereas the ESGE states that simethicone may improve colonic mucosa visibility (11, 12). A meta-analysis recently corroborated that using simethicone as an adjuvant may improve the quality of bowel cleansing and ADR during colonoscopy (24). It has also shown a lower bubble scale score and less intra-procedural use of simethicone in a recent RCT (25).

The combination of polyethylene glycol (PEG) with osmotic agents has also been studied. However, higher adverse events rates have been reported for sodium sulfate and sodium phosphate as vomit or electrolyte disturbances. Its use should therefore be restricted to selected patients (26).

Regarding stimulant agents, one meta-analysis that included six RCTs showed no significant differences in the quality of bowel cleansing between patients who received 2L PEG with bisacodyl or 4L PEG, although the adverse events were lower in the former group (27). A similar trend has been found in one RCT where better tolerability, satisfaction, and safety was reported in patients that received picosulfate, magnesium citrate, and bisacodyl than in subjects who received PEG (28).

Prokinetics agents such as metoclopramide is not related to better tolerability or quality of bowel preparation, so it is not routinely recommended for bowel preparation (11, 12).

A recent meta-analysis, which aimed to assess the efficacy of adjuvants in bowel preparation, reported a better cleansing quality in patients who received adjuvants, regardless of the regimen of administration. However, results should be interpreted with caution due to the high heterogeneity of the studies (17).

### Educational Tools

Patients must clearly understand the bowel preparation instructions. Indeed, the ESGE and ASGE recommend that patients should receive oral and written instructions in their native language and in plain language style (11, 12). However, up to 20% of patients fails to follow these recommendations (29). Therefore, several educational tools have been developed to improve comprehension and compliance.

Two meta-analyses showed significantly higher quality of bowel preparation and willingness to take the preparation in the group that received strategies focused on increasing knowledge and awareness compared with a standard-care group (18, 19) (Table 1), although the best tool to be implemented in clinical practice remains under discussion.

#### Additional Oral or Printed Explanation

Several RCT have shown that additional individualized oral information given by a trained nurse or physician improves cleansing quality (30–33). In addition, the use of cartoon visual aids, pictures, or booklets have shown contradictory results to improve the bowel cleansing quality in several RCT (34–37).

#### Audio-Visual Aids

Educational videos using plain language, illustrations, and video-clips could help patient comprehension. Two RCTs comparing conventional instructions with the same information plus an online videotape about the bowel preparation for colonoscopy reported significant higher rate of adequate bowel preparation assessed by BBPS (91.6 vs. 78.5%) (38) and global Ottawa score (5 vs. 4) (39) in the intervention group. Conversely, a more recent RCT comparing instructions by email and telephone calls vs. the addition of an educational video demonstrated no significant differences in the quality of bowel cleansing (40).

#### Mobile Phone Devices

Using this strategy, patients are re-educated in the bowel preparation while being reminded of the appointment. In two RCTs, patients who were assigned to receive a telephone call showed significantly higher rates of adequate bowel preparation (81.6 vs. 70.3%) (41) and better global BBPS scores (7.66 vs. 5.2) than patients who received conventional intructions (42). A similar trend was reported in an RCT where patients who received a telephone educational call or text message showed significantly better global BBPS scores than those who received conventional instructions (7.1, 6.8 vs. 6.3) (43).

#### Smartphone Applications and Social Media

These tools provide a source of health information as well as an easily accessible media platform for communication between patients and physicians. Two RCT that compared the use of a smartphone applications, including videos and pictures, to illustrate the bowel cleansing vs. conventional instructions showed a significantly higher rate of adequate bowel preparation in the intervention groups (44, 45).

The use of a social media application (WeChat) to provide information on bowel preparation also showed significantly better cleansing scores in one RCT compared to patients who received conventional instructions (82.2 vs. 69.5%) (46). However, although these educational tools seem to improve the quality of bowel cleansing, there is no evidence about which is the most cost-effective strategy. More research is needed in this field to assess the impact of the different strategies in specific populations, such as the elderly population or patients with a low educational level or socioeconomic status.

In summary, according to the ASGE and ESGE current guidelines (Table 2), to achieve optimal bowel preparation, it should be taken into account that: (1) 1 day of low-residue diet may be enough for non-selected patients who undergo a colonoscopy; (2) a split-dose cleansing regimen is the most effective method for patients undergoing colonoscopy in a morning schedule and split-dose or same-morning administration for afternoon colonoscopies; (3) the best quality of bowel cleansing is achieved when the colonoscopy is performed within 3–5 h after bowel preparation completion (11, 12).

**Table 2:** ESGE and ASGE recommendations for bowel preparation.

	**ESGE 2019 (12)**	**ASGE 2015 (11)**
Diet	Low fiber diet on the day preceding colonoscopy	Low-residue diet
Instructions	Enhanced instructions	Simple and easy to follow verbal counseling and written instructions that are simple in their native language
Adjuvants	ESGE suggests adding oral simethicone	
	ESGE does not suggest the routine use of prokinetic agents	ASGE recommends against the use of metoclopramide as an adjuvant
Timing	Split-dose bowel regimen for elective colonoscopy	Split-dose regimens
	For afternoon-shift colonoscopies a same-day bowel preparation is as an acceptable alternative to split dosing	For afternoon colonoscopies a same-day or split-dose regimen could be used
	The last dose of bowel preparation should be taken within 5 h of colonoscopy, and to complete it at least 2 h before the beginning of the procedure	A portion of the preparation should be taken within 3–8 h of the procedure
Laxatives	High or low volume PEG-based regimens as well as non-PEG-based agents that have been clinically validated for routine preparation	Bowel preparations should be individualized based on efficacy, cost, safety, and tolerability. This considerations should be balanced with the patient's overall health, comorbid conditions, and preferences
	In patients at risk for hydroelectrolyte disturbances, the choice of laxative should be individualized	Sodium phosphate and magnesium citrate preparations should not be used in the elderly or patients with renal disease or taking medications that alter renal blood flow or electrolyte excretion
Inadequate bowel preparation	To repeat the colonoscopy within 1 year, unless clinically contraindicated	To repeat the colonoscopy within 1 year
	Same-day or next-day colonoscopy after additional preparation (laxative or enema). The next regimen should be individualized according to the possible reasons for failure	To be considered for large-volume enemas or additional oral preparation before proceeding with colonoscopy. The next regimen should be more aggressive
Risk factors for inadequate bowel preparation	ESGE found insufficient data to recommend the use of specific predictive models for inadequate bowel preparation in clinical practice	ASGE suggest intensive education and more aggressive than standard bowel preparation

### Identification of Risk Factors for Inadequate Bowel Preparation

Most studies specifically designed to evaluate risk factors for inadequate bowel preparation have methodological flaws due to small sample sizes or a lack of a validated bowel preparation scale. Strong conclusions therefore cannot be drawn based on their results.

Sociodemographic features, such as age (elderly) (29, 47), sex (male) (47), relationship status (single) (29), or educational level (low) (48) have been suggested as predictive factors for inadequate bowel preparation; they might be linked to low compliance. Conversely, family history of CRC or personal history of polyps have been associated with better quality of bowel cleansing attributed to a higher motivation (29). Moreover, conditions associated with delayed colonic transit such as constipation (5, 49, 50), altered bowel anatomy due to previous abdominal or pelvic surgery (5) (including colorectal resection, mainly left resections) (5, 47, 51), drugs (tricyclic antidepressant, opioids or calcium antagonists) (5, 49), or comorbidities such as diabetes (5, 29, 47, 49) have been reported to be risk factors for inadequate bowel preparation. Physical activity restrictions due to obesity (47) and comorbidities have also been reported to be risk factors for poor bowel cleansing (5). Hospitalization status usually includes several of the previous conditions, such as a comorbidities, polypharmacy, delayed gastrointestinal motility, and physical activity restrictions. Thus, it is another risk factor for inadequate bowel cleansing (29, 49).

Colonoscopy-related factors also play an important role in the quality of bowel cleansing. These can include inadequate indication, a delay to the start of the colonoscopy of more than 5 h after completion of bowel preparation (48, 52), and previous history of inadequate bowel preparation, which is the most important risk factor for inadequate bowel cleansing (11).

Three prospective studies have developed a predictive model for inadequate bowel preparation. In a multicenter study including 2,811 colonoscopies, Hassan et al. (47) found that male sex, high body mass index, advanced age, history of colorectal resection, liver cirrhosis, Parkinson disease, and diabetes were risk factors for inadequate bowel preparation whereas a positive fecal occult blood test was a protective factor. They developed a predictive model that provided an area under the curve (AUC) of 0.63. However, in this study, only 12% of patients followed the currently recommended split-dose preparation regimen and a validated scale for bowel cleansing assessment was not used. Later, in a multicenter study, predictors of inadequate bowel preparation were assessed in 1,996 patients who received a high- or low-volume PEG split-dose. They found that an ASA score of ≥3, tricyclic antidepressants, opioids, diabetes, chronic constipation, history of abdominal and/or pelvic surgery, previous history of inadequate bowel preparation, and hospitalization were independent predictors of inadequate bowel preparation, and these were included in a predictive model providing an AUC of 0.77. However, this study had two main drawbacks as no standardized bowel preparation protocol was used and patients with a previous history of inadequate bowel preparation were included in the model (49). It is already known that the latter condition is enough to modify the bowel preparation protocol. To overcome these limitations, Gimeno-García et al. (5) assessed the risk factors for inadequate bowel preparation using the BBPS in 1,057 outpatients who received a same-day bowel preparation with high- or low-volume PEG or sodium picosulfate. Antidepressants, comorbidity, chronic constipation, and abdominal/pelvic surgery were independent predictors of inadequate bowel cleansing and were included in a prediction model providing an AUC of 0.70.

## Management of Patients at Risk of Inadequate Bowel Preparation

There is not enough evidence to recommend a specific strategy in patients with risk factors for poor bowel preparation. In recent years, several studies aimed to assess interventions to improve bowel cleansing in these patients.

### Personal History of Inadequate Bowel Preparation

The ESGE recommends to use irrigation pumps during colonoscopy or to schedule a new colonoscopy the following day with an additional preparation whereas the ASGE suggests repeating the procedure with a high-volume enema or additional oral preparation (11, 12).

In a prospective observational study of patients with a history of inadequate bowel preparation, an intensive bowel cleansing based on 3-day LRD, 4L PEG, and bisacodyl significantly improved bowel cleansing (53). These results were confirmed in a RCT where patients were allocated to receive 2L or 4L PEG along with 3-day LRD and bisacodyl. Indeed, patients in the former group showed a better rate of adequate bowel preparation without a significant difference in tolerance and neoplastic lesion detection rate. Furthermore, the highest benefit was achieved in patients who did not receive 4L PEG in the index colonoscopy (54).

### Diabetes Mellitus

Poor bowel cleansing in diabetic patients has been associated with constipation, nausea, and vomiting after the administration of bowel preparation due to a delayed gastrointestinal emptying. Another concern in diabetic patients is hypoglycemia, which could occur during bowel preparation. Therefore, to adjust the diet and antidiabetic drugs seems to be reasonable for their safety. One RCT assessed the quality of bowel preparation of a multifactorial strategy compared to the conventional strategy in patients who received a split-dose high-volume PEG solution. In the multifactorial strategy, patients received an educational intervention by a trained nurse who explained the bowel preparation, provided printed instructions, and adjusted the dose of antidiabetic agents for the procedure. A specific dietary plan consisting of a 4-day LRD and a liquid diet 8 h before the procedure was also provided. Conversely, patients allocated to the conventional strategy did not receive an educational intervention; they received written instructions that included recommendations for a 3-day LRD and 1-day liquid diet before the procedure. A significantly higher rate of inadequate bowel preparation was reported in the conventional compared to the multi-strategy group (20 vs. 7%) without significant differences in adverse event rates (4). Since it used a combination of different interventions, it was not clear what the effect of each strategy was by itself.

### Hospitalized Patients

Up to 34% of inpatients show inadequate bowel preparation, which entails rising costs due to repeated procedures and a longer hospitalization stay. Low socioeconomic status, drugs, ASA ≥3, nausea and vomiting, or older age have been reported as risk factors for poor bowel cleansing (55). A RCT that allocated inpatients to a higher volume PEG vs. low-volume PEG plus ascorbic demonstrated no significant differences in the quality of bowel preparation, but the latter was more acceptable (56).

### Chronic Constipation

Constipation (<3 bowel movements per week) increases the risk of inadequate bowel preparation five-fold. A recent RCT showed that bisacodyl plus simethicone and 2L PEG-citrate was more acceptable, increasing willingness to repeat the procedure and improved compliance when compared to 4L PEG without significant differences in the quality of bowel preparation (50). Another RCT reported a significantly higher rate of adequate bowel cleansing in patients who received 10 mg bisacodyl and 2L PEG-electrolytes compared with those who only received 2L PEG-electrolytes (88.7 vs. 61.2%) (57).

### History of Colorectal Resection

There is limited data regarding the best bowel preparation in patients with previous colorectal resection due to the exclusion of these patients from most studies aiming to assess the quality of bowel preparation. Based on expert opinions, high-volume bowel preparation has been recommended in these patients. Only one RCT that assigned these patients to receive a low- or high-volume split-dose preparation showed that the former was not inferior to the latter for adequate bowel cleansing quality, but tolerability was better in patients who received low-volume preparation (51).

#### Specific Devices

Several devices have been developed to improve the quality of bowel preparation during colonoscopy, such as JetPrep®, MedJet®, ClearPath®, PureVu®, or the water exchange, which are based on different irrigation pump methods. In another system called ColonoScoPrep® the colon is prepared by the infusion of warm water before colonoscopy. All of them have been reported to be safe and to improve the quality of bowel preparation, though RCTs are lacking (58–63). More evidence is thus needed to recommend them in clinical practice.

#### Future Directions

**Figure 1:**
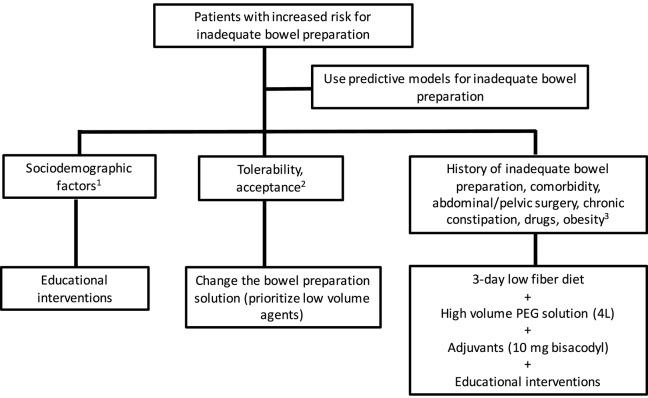
Recommendations to improve bowel cleansing in patients with increased risk for inadequate bowel preparation. (1) Sociodemographic features as elderly, male sex, single status, and low educational level are predictive factors for suboptimal bowel preparation. Enhanced education (additional audiovisual material or instructions) improves bowel cleansing quality in these individuals. (2) Patients with low tolerability or acceptance should be offered a different bowel cleansing solution, giving priority to low-volume agents. (3) Patients with a history of suboptimal cleansing, diabetes mellitus, abdominal, or pelvic surgery, chronic constipation, receiving tricyclic antidepressants, or obesity may need a combination of measures to improve to reach an adequate bowel preparation.

Poor bowel cleansing is a hodgepodge of situations as many conditions can cause it: poor compliance, poor tolerance, or a lack of efficacy. In such a way, we need different solutions for each specific condition. The next steps should therefore include studies that figure out the predictors of each of these conditions. Predictive models based on independent predictors of compliance or efficacy may help to better identify candidates suited for tailored strategies. RCT using different educational strategies or cleansing protocols should be focused on selected groups of patients, such as *a priori* known non-compliant patients or *a priori* known hard-to-prepare patients. In this sense, no study has evaluated a specific intervention for bowel preparation after applying predictive models. Thus, we suggest to follow the recommendations summarized in Figure 1 in an effort to optimize the bowel cleansing in patients with increased risk of inadequate bowel preparation.

## Conclusions

Based on this review we can make the following remarks: (1) low-volume preparation should be offered to non-selected patients who undergo a first-time colonoscopy or those who had an acceptable cleansing quality in the past with low-volume preparation protocols; (2) high-volume preparation should be reserved for those patients at risk of poor bowel preparation and predictive models may help to decide the best candidates for high-volume preparation; (3) adjuvants, such as bisacodyl, should be offered to patients with a past history of poor bowel preparation once poor compliance and tolerance have been ruled out; and (4) educational interventions focused on patient awareness and comprehension should be carried out in patients with past history of poor bowel preparation attributed to poor compliance.

## Author Contributions

All authors listed have made a substantial, direct and intellectual contribution to the work, and approved it for publication.

### Conflict of Interest

The authors declare that the research was conducted in the absence of any commercial or financial relationships that could be construed as a potential conflict of interest.
